# The pH-Insensitive Antimicrobial and Antibiofilm Activities of the Frog Skin Derived Peptide Esc(1-21): Promising Features for Novel Anti-Infective Drugs

**DOI:** 10.3390/antibiotics13080701

**Published:** 2024-07-26

**Authors:** Maria Rosa Loffredo, Floriana Cappiello, Giacomo Cappella, Elisabetta Capuozzo, Luisa Torrini, Fabiana Diaco, Yuanpu Peter Di, Maria Luisa Mangoni, Bruno Casciaro

**Affiliations:** 1 Laboratory Affiliated to Istituto Pasteur Italia-Fondazione Cenci Bolognetti, Department of Biochemical Sciences, Sapienza University of Rome, 00185 Rome, Italy; mariarosa.loffredo@uniroma1.it (M.R.L.); floriana.cappiello@uniroma1.it (F.C.); giacomo.cappella@uniroma1.it (G.C.); elisabetta.capuozzo@uniroma1.it (E.C.); bruno.casciaro@uniroma1.it (B.C.); 2 Department of Molecular Medicine, Sapienza University of Rome, 00185 Rome, Italy; luisa.torrini@uniroma1.it (L.T.); fabiana.diaco@uniroma1.it (F.D.); 3 Department of Environmental and Occupational Health, University of Pittsburgh, Pittsburgh, PA 15261, USA; peterdi@pitt.edu

**Keywords:** antimicrobial peptides, antibiotics, cystic fibrosis, acidic pH, biofilm, Gram-negative bacteria, infections

## Abstract

The number of antibiotic-resistant microbial infections is dramatically increasing, while the discovery of new antibiotics is significantly declining. Furthermore, the activity of antibiotics is negatively influenced by the ability of bacteria to form sessile communities, called biofilms, and by the microenvironment of the infection, characterized by an acidic pH, especially in the lungs of patients suffering from cystic fibrosis (CF). Antimicrobial peptides represent interesting alternatives to conventional antibiotics, and with expanding properties. Here, we explored the effects of an acidic pH on the antimicrobial and antibiofilm activities of the AMP Esc(1-21) and we found that it slightly lost activity (from 2- to 4-fold) against the planktonic form of a panel of Gram-negative bacteria, with respect to a ≥ 32-fold of traditional antibiotics. Furthermore, it retained its activity against the sessile form of these bacteria grown in media with a neutral pH, and showed similar or higher effectiveness against the biofilm form of bacteria grown in acidic media, simulating a CF-like acidic microenvironment, compared to physiological conditions.

## 1. Introduction

During host infections, bacteria encounter environments with acidic pH, such as the microenvironment of bacterial biofilm, the intracellular phagosomes and the airways of cystic fibrosis (CF) patients, for example. The genetic disease of CF is characterized by mutations in the gene encoding the CF Transmembrane Conductance Regulator (CFTR), an anion channel mainly expressed in the apical membrane of epithelial cells that controls the passage of chloride and bicarbonate ions [1]. The defective CFTR causes an irregular absorption of sodium and water molecules into the cells, producing thick secretions that obstruct airways, increasing the risk for frequent respiratory infections [2]. Furthermore, the loss of CFTR-mediated HCO_3–_ secretion generates an airway surface liquid (ASL) with reduced pH, contributing to the pathophysiology of CF lung disease [3,4]. Although the overall acidity of CF lungs is debated, several pieces of evidence in the ASLs of newborn CF pigs and differentiated human and porcine primary bronchial epithelial cell cultures have confirmed the acidic pH compared to non-CF controls [5,6,7,8]. Furthermore, the establishment of infections causes the acidification of the tissue microenvironment as a consequence of the inflammatory response of the host cells [9], and persistent infections are also considered initiators of the carcinogenesis process, which leads to the formation of a mostly acidic tumor microenvironment due to lactate secretion from anaerobic glycolysis [10,11]. Therefore, an effective strategy to prevent and/or treat infections is the administration of pH-insensitive antimicrobials. Although *Hemophilus influenzae* and *Staphylococcus aureus*, in the early stage, and *Pseudomonas aeruginosa* along the progression of age and disease, are the dominant infectious pathogens in CF, recent evidence highlighted the presence of several other microorganisms, such as *Acinetobacter baumannii* and *Klebsiella pneumoniae* [12,13,14,15,16]. The frequent exposure of these bacteria to antibiotics for the eradication of infections occurring from the first few months from birth has quickly led to the development of strains resistant to traditional therapies [17]. In addition, these strains can easily switch from a planktonic lifestyle to a more complex sessile form called biofilm, which is responsible for recalcitrant and persistent infections, mainly due to their intrinsic resistance to antibiotics [18]. Antibiotic molecules hardly reach bacteria that live in biofilms, because they are surrounded by an extracellular matrix composed of proteins, exopolysaccharides, lipids, nucleic acids, and other minor biomolecules, such as secondary metabolites [19]. In addition, many antibiotics act by inhibiting specific cellular processes that in bacterial biofilm cells are frequently slowed, making antibiotics ineffective [20]. For these reasons, new compounds active against biofilms and with different mechanisms of action are extremely necessary. In this regard, gene-encoded antimicrobial peptides (AMPs) of innate immunity represent an interesting class of molecules with expanding properties [21,22,23,24,25]. They possess (i) a broad spectrum of antimicrobial/antibiofilm activity; (ii) a primary bactericidal mechanism of action consisting of the membrane perturbation; and (iii) other biological features (i.e., wound healing capability) with respect to conventional antibiotics [26,27]. Many researchers have shown how pH variations can influence the bactericidal and bacteriostatic activity of antibiotics [28,29,30]; however, only a few studies have been reported on the effect of pH on AMP activity. We recently characterized a 21-residues-long derivative of the natural AMP esculentin-1a, namely Esc(1-21), with low cytotoxicity and potent in vitro and in vivo activity against Gram-negative bacteria [31,32,33,34,35,36,37,38]. In fact, it showed activity in mastitis-infected cows, in mouse models of acute *Pseudomonas*-induced pneumonia and sepsis and in mouse models of *Pseudomonas*-induced keratitis [39]. The primary mechanism of action of Esc(1-21) consists of the cytoplasmic membrane perturbation against both the planktonic and biofilm form of Gram-negative and Gram-positive bacteria [31,34,40]. Here, we first analyzed the effects of different pH (from 7.5 to 6.0) on the antibacterial activity of several AMPs, including Esc(1-21) and antibiotics. We then focused on the antibiofilm activity of Esc(1-21) against a panel of both reference and clinical isolates of Gram-negative bacteria, such as *A. baumannii*, *K. pneumoniae*, and *P. aeruginosa*. Overall, this research represents an important step to understanding how the infection environment can affect the efficacy of various antimicrobial agents, including AMPs.

## 2. Materials and Methods

### 2.1. Peptides

The peptides Esc(1-21), Bombinin H_2_, Temporin L, and LL37 were synthetically produced by Biomatik (Wilmington, NC, USA). Synthesis was carried out using a stepwise solid-phase technique and a standard F-moc protocol. Purification (to 95%) was performed via reverse-phase high performance liquid chromatography (RP-HPLC), and molecular mass was verified by electron spray ionization–mass spectrometry.

### 2.2. Materials

The 1-palmitoyl-2-oleoyl-sn-glycero-3-phosphoethanolamine (POPE) and 1-palmitoyl-2-oleoyl-sn-glycero-3-phosphoglycerol (POPG) were purchased from Avanti Polar Lipids (Alabaster, AL, USA). Sodium-dodecylsulfate (SDS) was obtained from Cambridge Isotope Laboratories (Tewksbury, MA, USA); carboxy fluorescein (CyF) and 3-(4,5-dimethylthiazol-2-yl)-2,5-diphenyltetrazolium bromide (MTT); colistin, ciprofloxacin and tobramycin were from Merck (Darmstadt, Germany). Cell Counting Kit 8 (CCK-8) was purchased from MedChemExpress (Monmouth Junction, NJ, USA).

### 2.3. Bacterial Strains and Eukaryotic Cells

For the microbiological assays, the reference strains were *A. baumannii* ATCC 19606, *P. aeruginosa* ATCC 27853, and *E. coli* ATCC 25922. Clinical *P. aeruginosa* strains were from a collection of the CF clinic at the Medizinische Hochschule of Hannover, Germany [41]; clinical MDR *A. baumannii* and *K. pneumoniae* were from the strain collection of Policlinico Umberto I (Sapienza, University of Rome). For MDR strains, the resistance profiles are reported in Table 1.

The human immortalized keratinocyte cell line HaCaT and the human type II alveolar epithelial cell line A549 were obtained from AddexBio (San Diego, CA, USA) and the American Type Culture Collection (ATCC, Manassas, VA, USA), respectively. Culture media were prepared by using Dulbecco’s modified Eagle’s medium supplemented with 4 mM or 2 mM L-glutamine (DMEMg); for HaCaT or A549 cells, respectively, 10% heat-inactivated fetal bovine serum (FBS), and 0.1 mg/mL of penicillin and streptomycin. The cells were maintained in 25 cm^2^ or 75 cm^2^ flasks in a humidified incubator at 37 °C and 5% CO_2_.

### 2.4. Antimicrobial Assays

Although there are distinct differences between the air and mucosal temperatures in the human respiratory tract, all in vitro analyses were conducted at 37 °C [42]. The minimum inhibitory concentrations of compounds were determined by the microdilution assay in a 96-well plate. Bacteria were grown in Luria–Bertani (LB) broth at 37 °C with gentle shaking until an optical density (O.D.) of 0.8 was reached (λ = 590 nm), and diluted in pH-adjusted 10% tryptic soy broth (TSB) in phosphate-buffer saline (PBS) (pH 7.5, 7.0, 6.5, and 6.0) at a concentration of 2 × 10^6^ cells/mL. Aliquots of 50 µL of this dilution were added to 50 μL pH adjusted 10% TSB diluted in PBS containing serial two-fold dilutions of the compounds. For antibiotics, concentrations ranging from 128 to 0.03 μg/mL were tested, while for AMPs concentrations ranging from 128 to 0.06 μM were used and then converted in μg/mL, for a better comparison. The controls were vehicle-treated cells. After an incubation time of 16–18 h at 37 °C, the MIC was defined as the lowest concentration, causing 100% visible inhibition of microbial growth. Each measurement was performed in triplicate [28].

The antibiofilm activity of peptide Esc(1-21) and tobramycin was evaluated against preformed biofilm in Luria–Bertani (LB) broth, as previously reported [43], after treatment in PBS at pH 7.5 and 6.5, for 2 h. In another set of experiments, biofilms were grown in an adjusted m63 medium, supplemented with 1 mM MgSO_4_, 25 µM FeCl_3_, 40 mM of D-glucose, and 4 mM of L-glutamine (pH 7.5 and 6.5) [28].

For both types of experiments, a microbial culture was grown in LB at 37 °C to an OD of 0.8 (λ = 590 nm) and then diluted in LB or pH adjusted m63 (pH 7.5 and 6.5) to a cell density of 1 × 10^6^ colony-forming units (CFUs)/mL.

Aliquots of 100 µL of this suspension were dispensed into the wells of a 96-multiwell plate and incubated for 20 h at 37 °C. After biofilm formation, planktonic cells were removed, and each well was washed twice with 150 µL of PBS to remove any non-adherent cells. After washing, each well was filled with pH-adjusted PBS (pH 7.5 and 6.5) supplemented with two-fold serial dilutions of peptide Esc(1-21) and tobramycin (from 1/4 MIC to 8× MIC), and the plate was then incubated for 2 h at 37 °C. Each well was washed with PBS twice. Biofilm viability was evaluated by adding 150 µL of MTT (0.5 mg/mL in Hank’s buffer) for 4 h (at 37 °C) and then the reaction was stopped by adding SDS at a final concentration of 5% *v/v*. The absorbance of each well was recorded at 570 nm using a microplate reader (Infinite M200; Tecan, Salzburg, Austria), and the percentage of biofilm viability was calculated with respect to the untreated samples.

### 2.5. Preparation of Large Unilamellar Vesicles (LUVs) and CyF Leakage Assay

Large unilamellar vesicles were prepared by dissolving POPE/POPG (7:3, molar ratio) in a 1:1 (*v/v*) chloroform/methanol mixture as previously described, with some modifications [32]. A rotavapor was used to guarantee the formation of a thin film by evaporating the solvents in a low-pressure evaporation. Liquid nitrogen was also used to allow for complete evaporation. Thereafter, the lipid film was hydrated with a CyF solution at a concentration that allowed for self-quenching, i.e., 30 mM, in 10 mM phosphate buffer containing 140 mM NaCl and 0.1 mM EDTA, pH 7.4 (buffer A).

To obtain LUVs, the liposome suspension was freeze–thawed (10 cycles) and then extruded through two layered polycarbonate membranes with 100 nm pores. Free CyF was eliminated by gel filtration using a 40 cm Sephadex G-50 column that had been equilibrated with buffer A at room temperature. The Stewart phospholipid test was used to quantify the final lipid content [44].

The peptide-induced membrane perturbation that causes CyF release from LUVs was observed by an enhancement in the fluorescence signal (excitation = 488 nm; emission = 520 nm) in pH-adjusted buffer A (pH 7.5 and 6.5) at 37 °C. CyF leakage from lipid vesicles (100 μM) following peptide addition at concentrations ranging from 50 to 0.5 μM (i.e., from 110 to 1.1 µg/mL) was observed for 30 min. Afterwards, 0.1% Triton X-100 was used to achieve complete dye release, after 5 min. Leakage (%) = 100 × (F1 − F0)/(Ft − F0) corresponds to the percentage of CyF leakage after peptide addition. F0 is the fluorescence of intact vesicles, and F1 and Ft are the intensities of the fluorescence attained by peptide and Triton X-100 treatment, respectively, at different time points, as indicated.

### 2.6. Cell Viability Assay

The viability of HaCaT and A549 cells was assessed using the CCK-8 assay. Briefly, 100 μL of cell suspensions (1 × 10^4^ cells) were added to each well of a 96-well plate and incubated for approximately 24 h at 37 °C and 5% CO_2_ [45,46]. Afterward, the medium was replaced by 100 μL of DMEMg at pH 7.5 or pH-adjusted DMEMg (pH 6.5), containing Esc(1-21) at different concentrations ranging from 4.3 μg/mL to 140 μg/mL, and the plate was incubated for 24 h. Then, the CCK-8 reagent was added to each well (1:10 dilution in DMEMg at pH 7.5 or 6.5), and after 1 h incubation, the absorbance of each well was measured at 450 nm using the microplate reader (Infinite M200; Tecan, Salzburg, Austria). The production of orange colored formazan dye is related to the number of living cells. The cell viability was expressed as a percentage with respect to the untreated control cells.

## 3. Results and Discussion

### 3.1. Antimicrobial Activity of AMPs and Antibiotics at Different pH Values, against Reference Gram-Negative Bacterial Strains

We initially studied the capability of Esc(1-21) and various AMPs in inhibiting microbial growth of three reference Gram-negative bacterial strains, compared to conventional antibiotics encompassing colistin, tobramycin and ciprofloxacin, in 10% TSB pH 7.5, as indicated in ref. [28]. The minimal inhibitory concentrations (MICs) are reported in Table 2.

**Figure 1:**
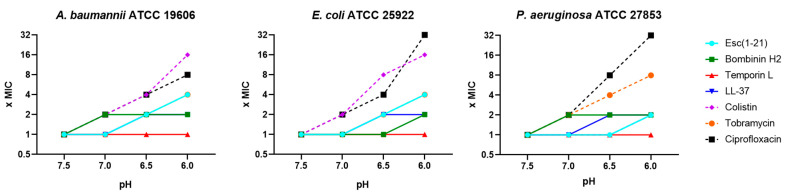
Variation in the MIC (expressed as fold-change in MIC value) of AMPs and antibiotics against *A. baumannii* ATCC 19606, *E. coli* ATCC 25922 and *P. aeruginosa* ATCC 27853, in 10% TSB at different pH values. The plotted data represent the modal values of three independent experiments.

The MIC values were then determined in media with increasing acidity (i.e., pH 7.5, 7.0, 6.5 and 6.0). As shown in Figure 1, the antimicrobial activity of all AMPs slightly decreased in parallel with the increasing acidity of the culture medium, as pointed out by the rising MICs, reaching a 4-fold higher value at pH 6. Conversely, the loss of activity of antibiotics was significantly more pronounced, with a 2–32-fold higher MIC at a pH lower than 7.5. These results are in line with what was observed by several authors; indeed, Kincses and colleagues tested erythromycin, ciprofloxacin, and gentamicin at different pH against clinical strains of *E. coli*, *P. mirabilis*, and *K. pneumoniae*, showing a similar loss in activity in acidic media [29]. A novel cephalosporin antibiotic that is among others indicated for the treatment of complicated urinary tract infections, named cefiderocol, showed similar results: the median MICs of this antibiotic were up to 50-fold higher at pH 5 than at pH 7 for *P. aeruginosa* isolates and 32-fold higher for *E. coli* and *K. pneumoniae* isolates [47]. Lin et al. tested ceftazidime, ciprofloxacin, and tobramycin at different acidic pH values against several *P. aeruginosa* strains and obtained a loss of activity in all cases, with the bacterial growth curves closer to those of the “no antibiotic treatment” controls [28]. With reference to AMPs, Abou Alaiwa and coworkers tested two well-known airway AMPs, i.e., human β-defensin-3 (HBD3) and LL-37. They found that lowering pH from 8.0 to 6.8 only attenuated the LL-37 killing of *P. aeruginosa*, while HBD3’s activity remained almost unchanged [48]. In some cases, AMPs have shown enhanced activity at low pH because of their basic properties, but this condition is related to the protonation of histidine at an acidic pH, an amino acid residue that is not present in the primary structure of Esc(1-21) [49,50,51].

**Figure 2:**
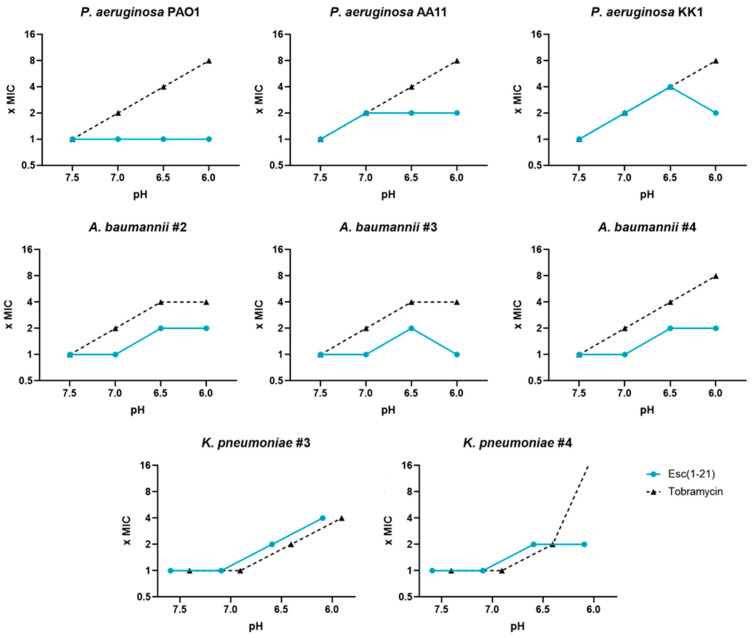
Variation in the MIC values of Esc(1-21) and tobramycin at different pH values against several Gram-negative strains, including reference and multidrug-resistant clinical isolates, in 10% TSB at different pH values. The data are the modal values from three independent experiments.

Subsequently, the activity of Esc(1-21) was evaluated against a panel of different Gram-negative strains, including reference and multidrug-resistant clinical isolates of *P. aeruginosa*, *A. baumannii*, and *K. pneumoniae*. Tobramycin was also included for comparison (Table 3 and Figure 2).

As indicated in Figure 2, the antimicrobial activity of Esc(1-21) was slightly influenced by the acidification of the medium, with a 2-fold higher MIC, except for *P. aeruginosa* KK1 and *K. pneumoniae* #3 against which a 4-fold increase in MIC was observed at more acidic pH (6.5 and 6.0, respectively). Interestingly, against *P. aeruginosa* PAO1 and *A. baumannii* #3, the MIC value of Esc(1-21) at pH 6.0 remained the same as that at pH 7.5. Similar results were obtained by Mohamed and collaborators, who analyzed several peptides against *P. aeruginosa* at an acidic pH: they observed a one-fold decrease in MIC at pH 5.5, except for the peptide named RR3, showing a four-fold decrease in MIC [52]. Tobramycin, on the other hand, showed an almost linear loss of activity with decreasing pH values, with 4-8-fold higher MIC than those obtained at pH 7.5, except for *K. pneumoniae* #4, against which the MIC was 16-fold higher. These results are in line with what was observed by several authors for tobramycin and other aminoglycosides [53,54].

### 3.2. Antibiofilm Activity of Esc(1-21) Compared to Tobramycin against Preformed Biofilm in LB Broth

**Figure 3:**
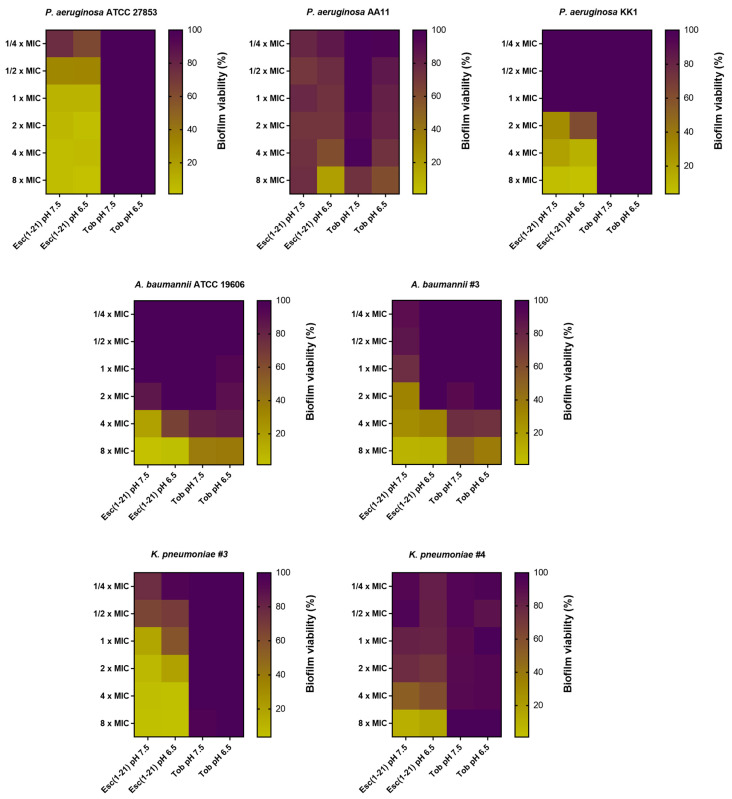
The effect of Esc(1-21) and tobramycin (Tob), against 20 h-preformed biofilms of several Gram-negative strains in LB and treated with the peptide/antibiotic for 2 h in PBS at two different pHs, i.e., 7.5 and 6.5. The colors represent the percentage of biofilm viability from 0% (yellow) to 100% (purple). Data were collected from three independent experiments.

During infections, Gram-negative bacteria colonize biological surfaces by creating matrix-enclosed aggregates, named biofilms. They are the cause of resistant and persistent infections that become the primary cause of death, especially in patients suffering from CF [55]. Furthermore, pH varies in the biofilm microenvironment: in the deep layers of a mature biofilm, an acidic pH exists due to the production of metabolites by bacterial cells, while the pH might be neutral at the biofilm periphery or in the close proximity of the water channels, the water-filled structures that, in mature biofilms, allow for the transport of nutrients, waste products and signaling molecules [56]. It is therefore crucial to investigate the activity of antibiotic compounds against the sessile form of bacteria at different pHs [57]. We previously reported the activity of Esc(1-21) against the biofilm form of *P. aeruginosa* [33,43]. However, we have no information on the effect of the peptide against biofilms in the presence of an acidic environment. For this reason, we tested Esc(1-21) at pH 6.5 against the biofilm form of three different strains of *P. aeruginosa* compared to tobramycin (Figure 3). In this set of experiments, biofilm was preformed in LB at pH 7.5 and then treated with the peptide/antibiotic for 2 h in PBS at two different pHs, i.e., 7.5 and 6.5. The main goal was to study the activity of the selected compounds in an acidic pH versus a neutral pH against biofilms grown under normal conditions. The antibiofilm activity was assessed by monitoring the formation of formazan crystals upon the reduction in the tetrazolium bromide salt by living bacterial cells. The higher the antibiofilm activity, the lower the formation of formazan crystals. The results showed that tobramycin did not possess any antibiofilm activity at all concentrations against most of the selected strains. A weak activity (~20–40% of biofilm reduction) was recorded against *P. aeruginosa* AA11 and *A. baumannii* strains, while no significant differences were observed at different pHs. On the contrary, Esc(1-21) was able to kill more than 50% of biofilm cells at concentrations equal to 4× and 8× MIC. The only exception was *P. aeruginosa* AA11 (~25% of biofilm killing). A potent activity was recorded against *P. aeruginosa* ATCC 27853, with more than 50% of killing also at concentrations of 2×, 1× and ½× MIC. The largest difference in antibiofilm activity at the different pHs was noted at lower peptide concentrations (from 2× MIC and below) versus *A. baumannii* #3. Notably, the activity of the peptide remained almost unchanged in the trend, apart from some small percentage variations at pH 6.5 against all the other strains. These results highlight the potential of AMPs as novel antimicrobial agents, considering the enhanced resistance to antibiotics of the bacteria embedded in biofilms [58]. Indeed, it has been reported that only a four-fold higher dosage of antibacterials is effective in killing bacteria in the biofilms, relative to the concentrations needed for the planktonic bacteria [59].

### 3.3. Antibiofilm Activity of Esc(1-21) Compared to Tobramycin against Preformed Biofilm Grown in m63 Medium at pH 7.5 and 6.5

**Figure 4:**
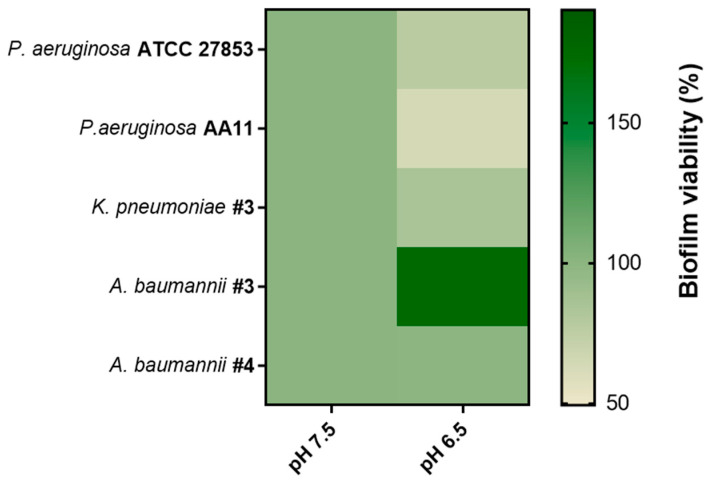
Percentage of biofilm viability of different Gram-negative strains in m63 at pH 6.5 with respect to pH 7.5 (set as 100%). The colors represent the percentage of biofilm viability from 0% (light green) to 100% (dark green). Data were collected from three independent experiments. The statistical analysis was conducted with the *t* test for all strains between the two different pH values: all differences were found to be significant (*p* < 0.05), with the sole exception of *A. baumannii* #4 (*p* = 0.765).

The acidic pH is known to influence microbial cell metabolism [60,61] to ensure pH levels compatible with cell viability. Indeed, bacteria have developed various mechanisms, such as (i) employing enzyme-catalyzed reactions that consume protons [62]; (ii) deploying reactions that produce basic compounds [63]; (iii) and eliminating protons from the cells at the expense of ATP consumption [64]. Gram-negative bacteria, such as the versatile pathogen *P. aeruginosa*, modify y gene expression to resist environmental stress. As an example, after exposure to a mildly low pH (5.0), *P. aeruginosa* shows an altered virulence-related phenotype with the induction of the expression of (i) two-component regulatory systems (i.e., *phoP*/*phoQ* and *pmrA*/*pmrB*); (ii) virulence genes (i.e., *pqsE* and *rhlA*); and (iii) lipid A remodeling genes (i.e., *arnT* and *pagP*) [65]. The acidic pH is also known to influence biofilm formation, which represents an interesting resistance mechanism [28,61]. A study by Hostacka et al. showed how different pHs of the media (i.e., 5.5, 7.5,8.5) modify the biofilm production of *P. aeruginosa*, *K. pneumoniae*, and Vibrio cholerae, with an increase in biofilm production observed with increasing pH [66]. The effect of pH on biofilm biomass was also evaluated against that of uropathogenic microorganisms, with similar results [67]. For this reason, we also investigated the capability of different strains (i.e., *P. aeruginosa* ATCC 27,853 and AA11, *K. pneumoniae* #3, *A. baumannii* #3 and #4) to form biofilm at two different pH conditions, pH 7.5 and 6.5 (Figure 4). To ensure a constant pH during biofilm formation, m63 medium was used, as reported by Lin et al. [28]. This medium was selected to maintain the pH consistency in simulating the CF-like acidic microenvironment.

**Figure 5:**
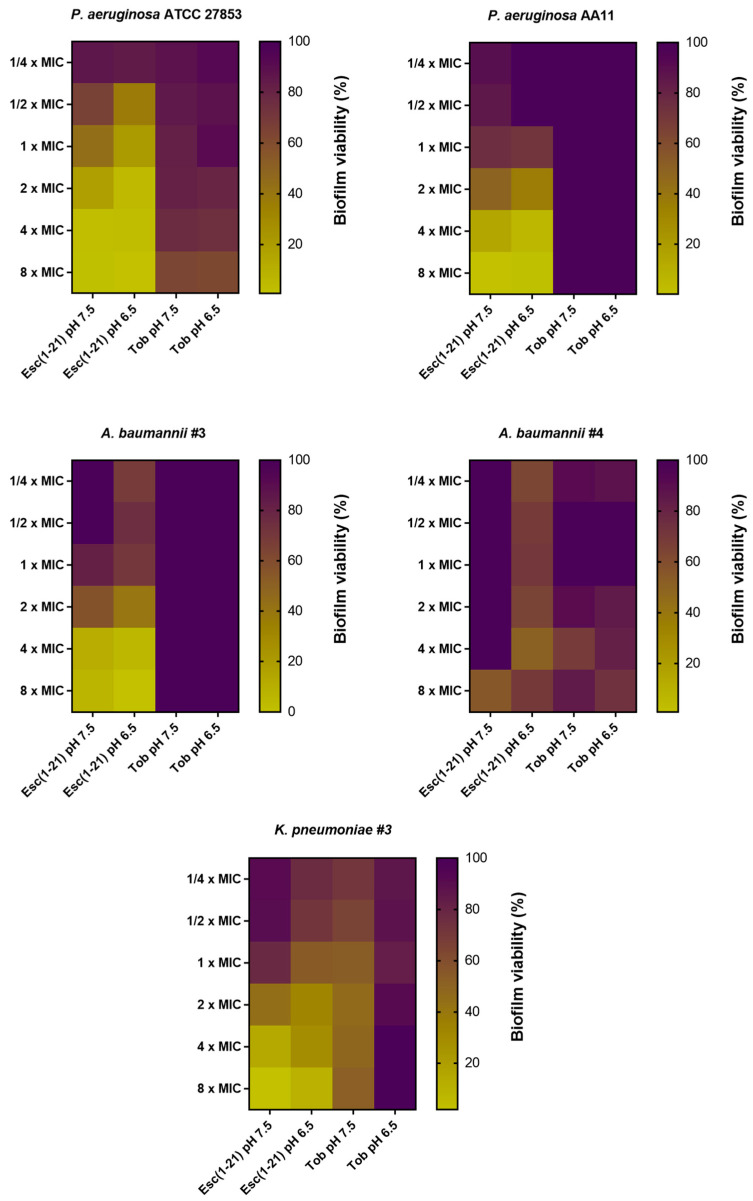
The effect of Esc(1-21) and tobramycin (Tob), against preformed biofilms of several Gram-negative bacterial strains in m63 medium at two different pHs, i.e., 7.5 and 6.5. Data were collected from three independent experiments.

As indicated in Figure 4, an acidic environment affected the biofilm formation of Gram-negative strains, aligning with what Lin et al. reported [28]. In fact, at pH 6.5, we observed a decreased biofilm viability of both *P. aeruginosa* and *Klebsiella* strains compared to pH 7.5. In contrast, an increased biofilm viability was found for *A. baumannii* #3, while no differences were recorded for *A. baumannii* #4. Then, Esc(1-21) was analyzed against these preformed biofilms for two hours in PBS at the corresponding pH, while tobramycin was used as the antibiotic control (Figure 5).

As shown in Figure 5, the activity of Esc(1-21) was enhanced against the biofilms of all the tested strains at pH 6.5. On the contrary, tobramycin had a significantly weaker activity at the two different pHs against the *P. aeruginosa* and *A. baumannii* strains, while activity against *K. pneumoniae* #3 was completely lost. For the first time, we explored the antibiofilm activity of the AMP Esc(1-21) in acidic pH, which is commonly one reason for the alteration of the activity of antibiotics [68].

### 3.4. Activity of Esc(1-21) at pHs 7.5 and 6.5 against Model Membranes of Gram-Negative Bacteria

The primary mechanism of action of Esc(1-21) is membrane perturbation with consequent cell death. To verify if this activity was influenced by pH variations, the peptide Esc(1-21) was tested at pH 7.5 and 6.5 on large unilamellar vesicles (LUVs) containing1-palmitoyl-2-oleoyl-sn-glycero-3-phosphoethanolamine (POPE)/(1-palmitoyl-2-oleoyl-sn-glycero-3-phospho-(1′-RAC-glycerol)(POPG), 7:3, mol–mol) to mimic the composition of the membrane of Gram-negative bacteria. The liposomes were loaded with a high concentration of Cyf to provoke self-quenching.

**Figure 6:**
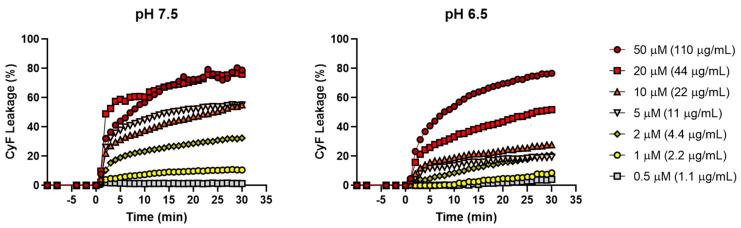
Kinetics of the effect of different concentrations of Esc(1-21) at pH 7.5 and 6.5, on the leakage of CyF encapsulated into POPE/POPG LUVs. LUVs were used at a final lipid concentration of 100 µM. Data points are the mean of three different experiments. SD is not shown for clarity.

After peptide addition (time 0), a fast and dose-dependent increase in the fluorescence signal was recorded (Figure 6), according to the membrane-perturbing mechanism of action of Esc(1-21) [31]. At pH 7.5., the percentage of CyF leakage was >60% at the high concentrations of 50 and 25 µM, while 20–50% of CyF leakage was recorded for the lowest concentrations. At pH 6.5, the peptide retained its membrane-perturbing activity, albeit to a lower intensity, with a CyF leakage of about 60% at 25 µM. These results are in agreement with those of Malik and colleagues, who analyzed the lysis levels of LUV by a linear reduced form of the frog-skin esculentin-2EM [69]. They demonstrated that the peptide had a general ability to induce the lysis of both the anionic and zwitterionic membranes, which was enhanced at a higher pH. In particular, in the case of phosphatidylethanolamine, used to simulate the membranes of Gram-negative bacteria, the peptide induced high levels of membrane lysis at the alkaline pH 8 (i.e., 83%), with only a slight reduction at the acidic pH 6, i.e., 60% [69].

### 3.5. Cytotoxic Activity of Esc(1-21) at Different pHs against Mammalian Cell Lines

**Figure 7:**
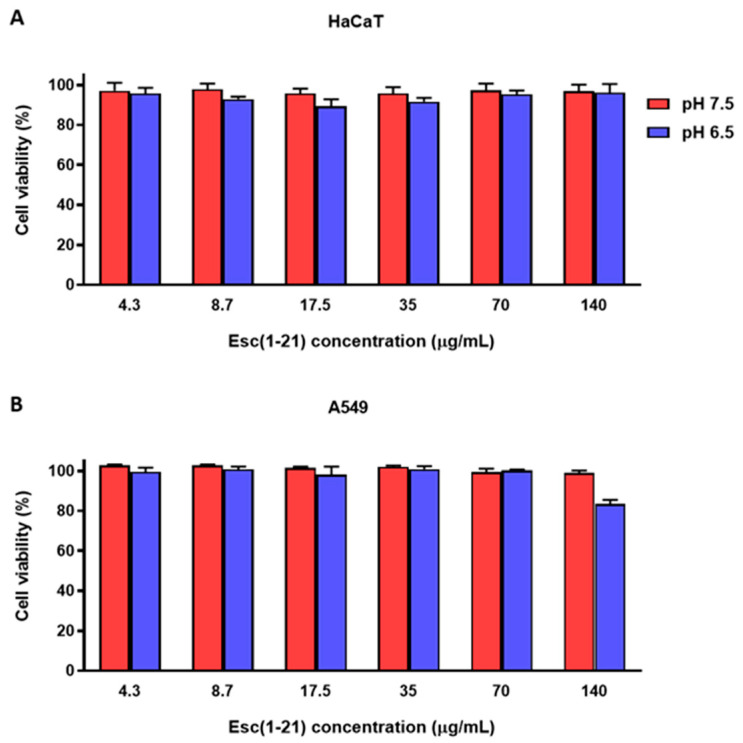
Viability of HaCaT (**A**) and A549 (**B**) cells after 24 h treatment with different concentrations of Esc(1-21) in DMEMg at pH 7.5 and 6.5. Cells not treated with peptides were used as controls. All data are the means of three replicates ± standard error of the mean (SEM). The statistical analysis was conducted with the *t* test for all concentrations between the two different pH values; all differences were found to be not significant (*p* > 0.05), with the only exception of 140 μg/mL against A549 cells (pH 7.5 vs. pH 6.5, *p* < 0.05).

We previously reported the safety profile of Esc(1-21) against several mammalian cell lines through the tetrazolium bromide reduction assay [43,70]. Here, the effect of Esc(1-21) on the viability of different mammalian cell lines, i.e., HaCaT and A549 cells, was studied in acidic conditions by the CCK-8 assay. As shown in Figure 7, we found that after 24 h treatment in the medium at pH 7.5 and 6.5, Esc(1-21) did not markedly reduce the percentage of viable cells at concentrations up to 140 μg/mL; only a slight reduction in cell viability (~20%) was recorded at the highest concentration tested, at pH 6.5 against A549 cells.

## 4. Summary and Conclusions

The increase in the number of MDR bacterial strains and the decrease in the development of new antibiotics are making antimicrobial resistance (AMR) one of the top 10 global public health threats facing humanity, according to the World Health Organization (WHO) [71]. Therefore, antimicrobial compounds not only have to overcome the obstacle of resistance development, but also have to reach the site of infection and maintain their biological activity. Acidic pH, such as that in CF airways, is one of the most serious causes of antibiotic treatment failure. In fact, in acidic microenvironments, bacteria modulate biofilm formation and become more resistant to antibiotics [72,73,74]. This, therefore, demands the identification of new antimicrobial agents with different mechanisms of action, that can retain activity in acidic conditions against both planktonic and biofilm forms of bacteria. In this work, the antimicrobial/antibiofilm activity of the AMP Esc(1-21) was explored when used at a pH lower than 7.5. It was found that when tested at pH 6.5, the peptide is practically insensitive to pH changes, since:

(i) It slightly loses activity (from 2- to 4-fold) against the planktonic form of Gram-negative bacteria. This is in contrast with what happens with traditional antibiotics that lose their activity up to 32-fold;
(ii) It retains antibiofilm activity against the sessile form of several Gram-negative bacteria grown in media with neutral pH; this does not happen for tobramycin, which is used as antibiotic control.
(iii) It shows a similar or higher antibiofilm activity against the sessile form of bacteria grown in acidic media as that found in infectious site microenvironments, including CF lung.

All these data reinforce the great potential of Esc(1-21) in the designing and developing of new antimicrobials to fight Gram-negative biofilm bacterial infections.

## Figures and Tables

**Table 1:** Resistance profiles of the tested MDR strains.

Strain	Resistance Profile
*K. pneumoniae* #3	AMI–CEFE–CEFO–CEFT–CIP–ERT–FOS–PIP/TAZ
*K. pneumoniae* #4	AMI–AMO/CLA–CEFE–CEFO–CEFT–CIP–ERT–PIP/TAZ
*A. baumannii* #2	AMI–AMO/CLA–CEFE–CEFO–CEFT–CIP–GEN–IMI–MER–PIP/TAZ–TRI/SUL
*A. baumannii* #3	AMO/CLA–AMP–CEFO–CIP–COL–ERT–GEN–IMI–PIP/TAZ–TRI/SUL
*A. baumannii* #4	AMO/CLA–AMP–CEFO–CIP–COL–ERT–GEN–IMI–PIP/TAZ–TRI/SUL

AMI, Amikacin; AMO, Amoxicillin; CLA, Clavulanic Acid; CEFE, Cefepime; CEFO, Cefotaxime, CEFT, Ceftazidime; CIP, Ciprofloxacin; GEN, Gentamicin; IMI, Imipenem; MER, Meropenem; PIP, Piperacillin; TAZ, Tazobactam; TRI, Trimethoprim; SUL, Sulfamethoxazole; COL, Colistin; ERT, Ertapenem; AMP, Ampicillin; FOS, Fosfomycin.

**Table 2:** Minimal inhibitory concentrations (MICs) of various AMPs and antibiotics against three representative Gram-negative bacteria: *A. baumannii* ATCC 19606, *E. coli* ATCC 25,922 and *P. aeruginosa* ATCC 27853, in 10% TSB at pH 7.5.

	MICs (µg/mL)		
Compound	*A. baumannii* ATCC 19606	*E. coli* ATCC 25922	*P. aeruginosa* ATCC 27853
Esc(1-21)	4.3	4.3	8.7
Bombinin H2	15.3	61.3	245
Temporin L	3.25	6.5	6.5
LL-37	9.0	9.0	4.5
Colistin	0.5	0.5	0.25
Tobramycin	2.0	1.0	0.125
Ciprofloxacin	0.25	0.25	0.125

The data are the modal values from three independent experiments.

**Table 3:** MICs of Esc(1-21) and tobramycin against several Gram-negative strains, including reference and multidrug-resistant clinical isolates, in 10% TSB, pH 7.5.

	MICs (µg/mL)	
Strain	Esc(1-21)	Tobramycin
*P. aeruginosa* PAO1	8.7	0.25
*P. aeruginosa* AA11	2.2	0.25
*P. aeruginosa* KK1	17.5	0.125
*A. baumannii* #2	2.2	16
*A. baumannii* #3	2.2	16
*A. baumannii* #4	2.2	16
*K. pneumoniae* #3	8.7	32
*K. pneumoniae* #4	8.7	64

The data are the modal values from three independent experiments.

## Data Availability

Data reported in this work are available upon request to the corresponding authors.
